# Comparative Analysis of Large Language Models in Emergency Plastic Surgery Decision-Making: The Role of Physical Exam Data

**DOI:** 10.3390/jpm14060612

**Published:** 2024-06-08

**Authors:** Sahar Borna, Cesar A. Gomez-Cabello, Sophia M. Pressman, Syed Ali Haider, Antonio Jorge Forte

**Affiliations:** 1 Division of Plastic Surgery, Mayo Clinic, Jacksonville, FL 32224, USA; 2 Center for Digital Health, Mayo Clinic, Rochester, MN 55905, USA

**Keywords:** large language models, ChatGPT-4, Gemini, artificial intelligence, plastic surgery, physical exam

## Abstract

In the U.S., diagnostic errors are common across various healthcare settings due to factors like complex procedures and multiple healthcare providers, often exacerbated by inadequate initial evaluations. This study explores the role of Large Language Models (LLMs), specifically OpenAI’s ChatGPT-4 and Google Gemini, in improving emergency decision-making in plastic and reconstructive surgery by evaluating their effectiveness both with and without physical examination data. Thirty medical vignettes covering emergency conditions such as fractures and nerve injuries were used to assess the diagnostic and management responses of the models. These responses were evaluated by medical professionals against established clinical guidelines, using statistical analyses including the Wilcoxon rank-sum test. Results showed that ChatGPT-4 consistently outperformed Gemini in both diagnosis and management, irrespective of the presence of physical examination data, though no significant differences were noted within each model’s performance across different data scenarios. Conclusively, while ChatGPT-4 demonstrates superior accuracy and management capabilities, the addition of physical examination data, though enhancing response detail, did not significantly surpass traditional medical resources. This underscores the utility of AI in supporting clinical decision-making, particularly in scenarios with limited data, suggesting its role as a complement to, rather than a replacement for, comprehensive clinical evaluation and expertise.

## 1. Introduction

In the United States, nearly 12 million adults experience a missed or incorrect diagnosis, constituting about 5.08% of outpatient cases, or roughly one in every 20 adults annually [1]. In hospital settings, at least 0.7% of adult cases involve a misdiagnosis, often concerning common diseases [2]. Notably, the most critical misdiagnoses involve infections, vascular events, and cancers, averaging 9.7% of such cases [3,4]. Accurate diagnosis is crucial in delivering effective healthcare services and represents the primary responsibility of physicians [5,6]. The diagnosis and management processes are essential in assessing the quality and safety of the healthcare system [7], and research indicates that approximately 0.8% of hospital admissions over two years experience medication errors [8]. Various factors contribute to these practical errors, including the involvement of multiple clinicians and the complexity of healthcare processes [9]. While a significant proportion of these errors occur during the test ordering phase, inadequate history-taking and physical examination are responsible for about 10% of these mistakes [10]. One contributing factor to these errors is delayed access to healthcare professionals [11], often due to geographical constraints [12]. Additionally, lower health literacy can prevent individuals from seeking necessary healthcare, especially when they lack understandable and communicative resources [13]. Moreover, even remote healthcare systems, though beneficial, are not immune to errors, as mistakes can arise at any stage of remote patient management [14].

Clinical decision tools, particularly those augmented with Artificial Intelligence (AI), significantly enhance patient management by providing high-confidence, low-error decision support. AI’s capability to rapidly analyze vast datasets empowers healthcare professionals to make more accurate decisions [15,16,17,18,19]. Utilizing Machine Learning (ML) and Natural Language Processing (NLP), these tools excel at mining medical data. This approach is beneficial for preventive medicine and essential in the diagnosis and patient management, effectively managing the ever-growing volume of health data [17,20].

Large Language Models (LLMs) like OpenAI’s Generative Pre-Training Transformer 4 (ChatGPT-4) [21] and Google Gemini [22], integral to artificial intelligence, leverage deep learning and NLP to generate responses resembling human conversation [23,24]. Recent advancements have increasingly highlighted the potential of LLMs in enhancing patient management, mainly due to their user-friendly nature and ability to overcome language barriers [25,26]. Research efforts have been made toward developing criteria for evaluating the quality of LLM-provided rationales in clinical contexts, with a focus on their consistency, specificity, and alignment with human reasoning processes [27]. These models have demonstrated proficiency in delivering high-quality management information, particularly for specific types of cancerous tumors [28]. Additionally, their use as diagnostic tools has been explored, with notable examples in common and rare diseases and ENT (Ear, Nose, and Throat) cases [29,30]. Furthermore, studies have examined LLMs’ capabilities in generating differential diagnoses, with evidence suggesting that clinicians supported by LLMs can provide more accurate and comprehensive differential diagnosis lists compared to traditional methods [5]. This indicates a promising role for LLMs in assisting physicians with complex diagnostic challenges, underscoring their utility beyond that of conventional search engines.

A study compared ChatGPT’s diagnosis accuracy in rheumatology to a physician’s, finding ChatGPT more effective in correctly diagnosing rheumatologic conditions, particularly in distinguishing inflammatory from noninflammatory diseases [31]. While studies show that ChatGPT aids in tailored treatment, reducing healthcare costs [26], no research has yet explored the impact of physical examination data on the performance of LLMs. This gap is significant because LLMs currently lack the capability to assess or access such data. Physical examination information plays a crucial role in physicians’ influencing patient management outcomes. However, it remains unclear how the absence of this data might influence the diagnostic and management decisions made by LLMs. In this study, we aimed to evaluate and contrast the capabilities of the leading large language models, ChatGPT-4 and Google Gemini, in delivering diagnostic and management guidance for typical emergency scenarios in plastic and reconstructive surgery. We aimed to evaluate the capabilities of publicly available large language models, excluding those enhanced with retrieval-augmented generation techniques. We investigated their performance both with and without the inclusion of physical examination data. In Section 2, we describe the methodology used to evaluate the diagnostic accuracy and management proficiency of two language models, ChatGPT-4 and Gemini, within the context of emergency plastic surgery scenarios. Section 3 presents the results of the study, including statistical analyses and performance comparisons between the models. Section 4 offers a detailed discussion of the findings, addressing diagnostic accuracy, management performance, and limitations of the study. Finally, Section 5 concludes with a summary of key insights and potential future research directions.

## 2. Materials and Methods

In this study, we evaluated the diagnostic accuracy and management proficiency of two language models, ChatGPT-4 and Gamini, in the context of emergency plastic surgery scenarios. We developed 30 medical vignettes based on patient cases, initially excluding physical exam data, and subsequently enhanced these vignettes by incorporating the physical exam information. The vignettes encompass a range of topics, including hand surgery, burn, lip, ear, and eyelid lacerations, skull fractures, sternal wounds, facial hematoma and nerve injuries, parotid duct injuries, mandibular fractures, nasal fractures, nasal septal hematoma. These scenarios were carefully designed by an expert plastic surgeon to cover a diverse spectrum of emergency conditions commonly encountered in plastic surgery. We initially presented clinical vignettes lacking physical examination details to two language models, ChatGPT-4 and Gemini, requesting diagnostic interpretations. Subsequently, in separate sessions, we re-introduced these vignettes to each model, this time seeking management advice. Following this, we provided the same vignettes once more, now including physical examination data, and asked for both diagnosis and management in distinct sessions for each language model. Each language model was tasked with responding to these vignettes, simulating a real-world medical decision-making process (Table 1).

Overall, 240 questions were asked, and each response provided by ChatGPT-4 and Gemini was meticulously evaluated for clinical appropriateness by three qualified medical professionals, utilizing ‘*Plastic Surgery Emergencies: Principles and Techniques, Second Edition*’ by Bullocks et al. [32] as the standard reference. We employed a three-point Likert scale to quantify the alignment of each response with the textbook guidelines. We assigned a score of 1 for cases with no diagnosis or an incorrect diagnosis, a score of 2 if the correct diagnosis was included in the Differential Diagnosis (DDx), and a score of 3 when the primary diagnosis was correctly identified.

For management responses, a score of 1 indicated low alignment, 2 indicated moderate alignment, and 3 signified high alignment with the established clinical standards. We asked three healthcare professionals to independently score the AI model’s answers. In case of any scoring discrepancies, the assessors discussed reaching a consensus, ensuring a unified and agreed-upon evaluation.

Furthermore, we thoroughly analyzed the response distribution for each language model. The frequency of responses within each score category was recorded, both with and without the incorporation of physical exam data, and presented as a percentage of the total responses. We assessed overall performance and calculated key statistical measures for each category, including the median and the mode.

**Figure 1:**
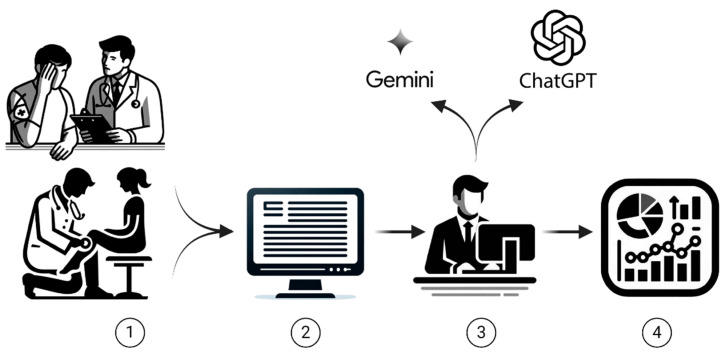
[21] (**1**) We created 30 patient vignettes based on typical emergencies in plastic surgery, enhancing each with detailed physical exam findings for comprehensiveness. (**2**) Two sets of prompts, each asking for diagnosis and management, were developed and (**3**) presented separately to two large language models (LLMs), ChatGPT-4 and Gemini. Each LLM was tested in distinct sessions to ensure independent assessments. (**4**) The responses were recorded and later analyzed to evaluate and compare the effectiveness of the LLMs in handling medical scenarios.

For statistical analysis, the Wilcoxon rank-sum test was used to compare the rankings of Likert scores across different scenarios for each model and between the models themselves. This included comparing performance in diagnosis and management tasks with and without physical exam data. The Wilcoxon rank-sum test was chosen to robustly assess any statistically significant differences in the models’ performance, with a conventional significance level of 0.05 guiding the interpretation of results. Figure 1 and Table 2.

## 3. Results

### 3.1. ChatGPT-4 and Gemini Performance

Statistical analysis was performed to assess ChatGPT-4 and Gemini’s diagnostic and management performance using the Wilcoxon rank-sum test. For ChatGPT-4, the median score stood at 3 for diagnosis and 2 for management, irrespective of physical exam data, with modes aligning with these medians. The respective Wilcoxon W values were 400.5 for diagnosis and 424 for management, with non-significant *p*-values of 0.24 and 0.62. Similarly, Gemini showed consistent median scores of 3 in diagnosis and 2 in management, with Wilcoxon W values of 378 and 464.5 and *p*-values of 0.23 and approximately 0.8, indicating no significant impact from the inclusion or exclusion of physical exam data. The *p*-values remained above the 0.05 significance threshold for both models and all scenarios, affirming the non-significant difference in median ranks, as detailed in Table 3, Table 4 and Table 5.

### 3.2. Integrated Analysis of ChatGPT-4 and Gemini

Diagnostic Performance: Both models displayed robust diagnostic capabilities, with ChatGPT-4 achieving higher accuracy in more cases. The integration of their results suggests that ChatGPT-4’s detailed diagnostic rationales and Gemini’s solid grasp of clinical situations can be complementary, particularly in scenarios with limited physical examination data. ChatGPT-4 showed statistically significantly higher performance in diagnosis both with and without physical exam data (*p*-values of 0.030 and 0.023, with Wilcoxon W of 561 and 573, respectively). The accuracy percentages for diagnosis were 90% without physical exam data and 100% with physical exam data for ChatGPT-4, compared to 73.33% and 86.67% for Gemini, as detailed in Table 4. The accuracy percentages are calculated by considering the sum of scores 2 and 3 as accurate.

**Figure 2:**
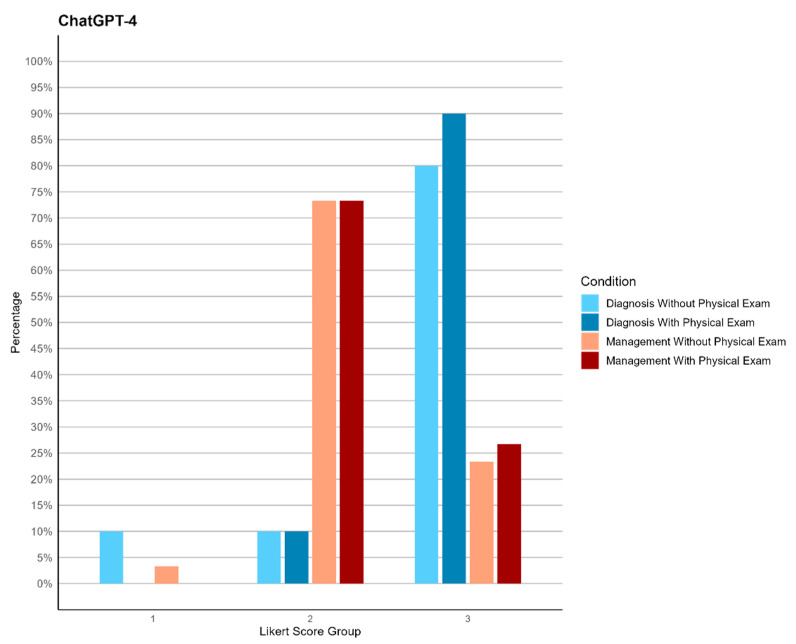
Bar plot of ChatGPT-4’s performance in diagnosis and management, before and after receiving physical exam data.

**Figure 3:**
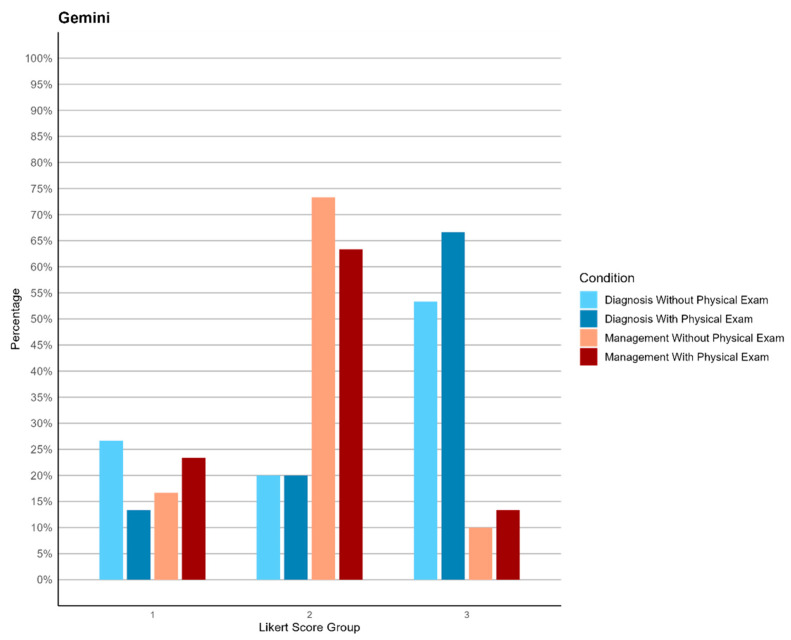
Bar plot of Gemini’s performance in diagnosis and management, before and after receiving physical exam data.

Management Performance: In management tasks, ChatGPT-4 provided more detailed and clinically aligned recommendations, whereas Gemini’s responses, while accurate, were less specific. The combined use of both models could enhance clinical decision-making by leveraging ChatGPT-4’s detailed management plans and Gemini’s initial assessment capabilities. The significant differences in management performance were evidenced by *p*-values of 0.048 without physical exam data and 0.014 with physical exam data, with Wilcoxon W of 554 and 587, respectively. The accuracy percentages for management were 96.67% without physical exam data and 100% with physical exam data for ChatGPT-4, compared to 83.33% and 76.67% for Gemini, as detailed in Table 4, Figure 2 and Figure 3.

**Figure 4:**
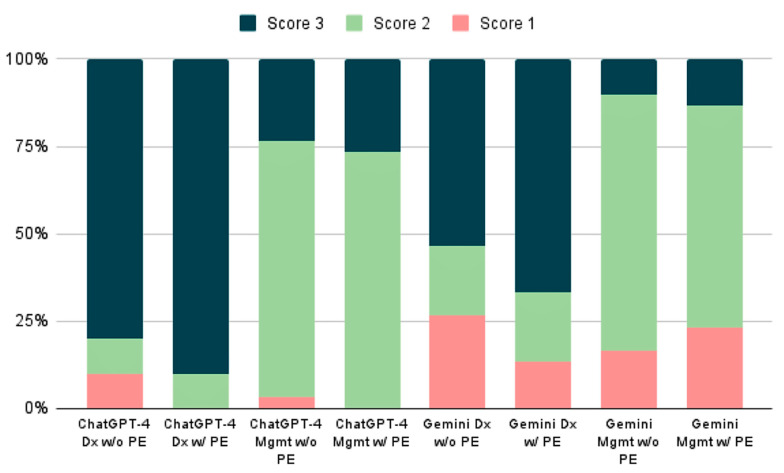
Stacked bar chart comparing Gemini and ChatGPT-4 scores on diagnosis and management prompts. Abbreviations: Dx (Diagnosis), Mgmt (Management), W/PE (with a physical exam), W/O PE (without a physical exam).

These findings underline consistent and statistically significant differences in how each model performs, adhering to a 5% significance level for diagnostic and management tasks (Figure 4).

## 4. Discussion

### 4.1. Diagnostic Accuracy with and without Physical Examination

Multiple studies have explored the application of LLMs, particularly ChatGPT-4, in providing accurate diagnoses from various vignettes and scenarios [33]. Some of these studies have employed actual case reports for their analysis [24], while others have utilized questions from comprehensive databases like the USMLE Step 1 [33].

In our study, in several cases, Gemini successfully identified the accurate differential diagnosis, indicating a solid grasp of the clinical situations described. This proficiency aligns with standard textbook guidelines, yet Gemini frequently refrained from offering a definitive diagnosis in the absence of a physical examination. Similarly, ChatGPT-4 exhibited competence in providing differential diagnoses with logical reasoning for each option. Notably, in several situations, the primary differential diagnosis suggested was accurate, underscoring a fundamental understanding of the clinical contexts.

In a study exploring ChatGPT-4’s ability to generate differential diagnoses from New England Journal of Medicine (NEJM) case reports, researchers employed four prompt generation methods: Narrative Prompt Generation (NARR), Phenotypic Feature Prompt Generation (PHENO-R and PHENO-C), and Manual/HPO Prompt Generation (MAN-HPO). The research assessed ChatGPT-4’s consistency across two model versions, noting performance variations. Notably, enhanced responses were observed when including medical history, family history, comorbidities, and lab results in MAN-HPO prompts, yet these did not reach the accuracy of complete narrative prompts from the NEJM cases [24]. Conversely, various studies have yielded mixed findings. One investigation highlighted ChatGPT-3’s superiority over Isabel [34] and found the superior quality of GPT in providing DDx for ophthalmic scenarios, emphasizing its potential value for primary care providers [35] in generating differential diagnoses for eye-related conditions, underscoring its utility for primary care. Other research indicated that ChatGPT-4’s effectiveness in diagnosis is more pronounced in a multiple-choice context. This implies that its practicality in real-world medical scenarios may be more limited outside of structured formats. An ablation study revealed that even slight alterations in question framing significantly affect ChatGPT’s response quality and accuracy. This finding diverges from our results. However, the nature of information omitted or included in prompts is crucial: past medical history might be critical for diagnosing chronic conditions but less so for acute, plastic surgery-related issues [33]. However, the lack of physical exam data in our study led to limitations in ChatGPT’s diagnostic specificity. For instance, ChatGPT could recognize the presence of a skull fracture but was unable to determine the specific type, like a frontal sinus fracture. This limitation highlights the critical role of physical examination in ensuring accurate and precise diagnoses.

In some instances, Gemini’s diagnostic accuracy was found to be lower, either suggesting diagnoses unrelated to the patient’s issues or ranking the correct diagnosis lower in its list of possibilities. ChatGPT, on the other hand, consistently avoided these issues. In 14 instances, Gemini explicitly stated its limitations as a language model, unable to offer diagnoses or management strategies. This limitation was evident even when Gemini could identify the diagnosis but refrained from advising on management or vice versa. This represents a statistically significant difference in diagnostic capability between the two tools, with ChatGPT-4 consistently receiving higher scores for the appropriateness of its responses, both before and after incorporating physical exam data. Gemini frequently requested additional information, particularly physical examination details. However, the quality of its responses did not significantly improve upon receiving this extra data.

Gemini and ChatGPT-4 showed diagnostic enhancements upon incorporating physical exam data. Specifically, ChatGPT-4’s diagnostic accuracy improved from 24 (80%) to 27 (90%) cases, while Gemini’s accuracy rose from 16 (53.33%) to 20 (66.67%). This underscores the value of physical exam details in clinical decision-making, as AI tools provide more precise and applicable diagnoses. ChatGPT-4, notably, was adept at recognizing the exact locations of medical issues. However, limitations were observed; both systems sometimes offered irrelevant differential diagnoses, expressed uncertainty, or lacked depth in conveying the gravity or implications of the conditions. This highlights a potential shortfall in their capability to fully adapt and interpret complex clinical data. Interestingly, while adding physical examination information enriched the detail in the LLMs’ responses, it did not lead to a statistically significant enhancement in response quality. Notably, ChatGPT exhibited a more accurate diagnostic performance than Gemini. However, the improvement in response quality post-receipt of additional information was parallel for both language models.

### 4.2. Performance in Management Orders with and without Physical Examination

Research indicates that while LLMs can offer management strategies that physicians might miss, they also have the potential to deliver entirely incorrect responses in certain situations [36,37]. Our study reveals that in comparison to standard textbooks, LLMs tend to generate less detailed and specific management plans. This is particularly evident in complex cases such as open fractures, burns, facial nerve injuries, and mediastinitis and reconstructive techniques. Key aspects where LLMs fall short include prescribing precise antibiotic regimens, determining criteria for specialist referrals, formulating fluid resuscitation strategies, and implementing advanced surgical methods.

While Gemini and ChatGPT-4 both offer initial management strategies suitable for emergency room contexts, they tend to lack depth and specificity. They provide a broad outline, emphasizing immediate actions such as pain relief and wound care, but fall short in detailing specific treatments and procedures. This mirrors ChatGPT-4’s approach, which focuses on general management steps and immediate interventions like pain management, infection prevention, and subsequent referrals, aligning with the basic principles found in the textbook. However, both Gemini and GPT answers miss crucial specifics. In contrast, the textbook excels by providing more comprehensive guidance, including detailed antibiotic protocols, wound management techniques, and surgical considerations.

ChatGPT-4 occasionally offered management suggestions in addition to diagnoses, even when these recommendations were not directly requested. This approach demonstrates a proactive stance, yet it may also indicate a deviation from the specific focus of the question asked.

After receiving the physical exam data, the management plans generated by the Gemini tool exhibited greater detail and relevance. However, they exhibited critical deficiencies, notably in specifying the administration routes and selecting appropriate antibiotics. This highlighted a limitation in the AI tool’s capacity to provide all-encompassing management strategies. The management plans from Gemini corresponded closely with textbook guidelines in terms of immediate actions and general care advice. Yet, they lacked the necessary depth in areas like wound care, antibiotic treatment for open fractures, and precise surgical procedures.

In this context, evaluating LLMs as a prominent AI tool used by both medical healthcare providers and individuals outside of healthcare is particularly beneficial. This is especially true in remote and underserved areas where access to healthcare professionals is limited or in situations where immediate access is impeded by factors like distance or the absence of specialist plastic surgeons. We specifically chose emergency cases in plastic surgery to assess the LLMs’ ability to quickly provide quality diagnoses and management advice. By eliminating the physical exam component, we aimed to determine whether, in the absence of a professional plastic surgeon, insufficient time, the inability to conduct a full physical exam, and the AI’s inherent inability to perform physical examinations, the LLMs could still deliver high-quality responses in patient management. Our study demonstrates that while there are differences in the quality of information provided by the LLMs, these differences are not statistically significant.

Overall, these AI tools exhibit a strong basic grasp of emergency plastic surgery scenarios. However, they lack the detailed diagnostic and treatment insights typically found in standard medical textbooks. As AI progresses, it becomes increasingly clear that it can enhance, rather than supplant, human decision-making in healthcare.

### 4.3. Limitations

This study’s limitations are primarily due to the scope and methodology employed. The use of only 30 questions restricts the ability to fully encompass the vast array of possible medical scenarios, potentially impacting the representativeness of the findings. Variability in the format and depth of the vignettes poses another challenge, as different prompts and the varying amounts of information they contain can significantly influence the AI’s responses. Grouping the results by specific types of trauma or problem locations could offer more targeted insights, yet this approach was not utilized, potentially affecting the depth of analysis. Furthermore, the subjective nature of interpreting the AI’s responses compared to textbook standards introduces an element of personal bias, which might impact the evaluation of the information’s relevance and thoroughness. The applicability of the study’s findings is limited, as they are based on responses from specific AI tools (Gemini and ChatGPT-4) under controlled conditions, which might not accurately reflect performance across different AI platforms or in varied clinical settings. Lastly, this study did not explore the inner workings of the LLMs and focused on evaluating their performance for the specific task of diagnosing and managing emergency plastic surgery scenarios.

## 5. Conclusions

In our study comparing ChatGPT-4 and Gemini across 30 emergency plastic surgery vignettes, ChatGPT-4 demonstrated superior performance in both diagnosis and management, regardless of whether patient physical exam data was included. Without physical exam data, ChatGPT-4’s proficiency was evident, offering accurate differential diagnoses and general management strategies, although it lacked the detail and specificity found in medical textbooks. Incorporating physical exam data improved diagnoses and management plans, but the level of detail still fell short compared to textbooks, highlighting the value of integrating AI with extensive clinical data and expertise for optimal outcomes. The conservative approach of AI tools in scenarios without physical exam data underscores their limitation in complex emergencies. Thus, while these AI tools are promising adjuncts in clinical decision-making, particularly when combined with comprehensive clinical examination and expertise, they are supplements to, rather than replacements for, the nuanced guidance offered by medical textbooks in emergency plastic surgery. Further investigation is needed to evaluate the efficacy of a retrieval-augmented generation strategy.

## Figures and Tables

**Table 1:** Examples of sequential prompts given to language models.

Examples of Prompts Provided to LLMs	
First Prompt	I am a licensed plastic surgeon, I will give you a scenario about a patient coming to the hospital, and I want you to tell me the next step in management that should be done for the patient (if there is a need for specific type of imaging, antibiotic therapy, or surgery, mention them).
Second Prompt	A 36-year-old woman presents to the emergency room, reporting persistent pain and muscle weakness in her left forearm, which began two hours ago. She has a history of hand trauma that occurred one day prior. During the physical examination, the patient experiences exacerbated pain with passive muscle stretching and active flexion in the forearm. Sensory deficits are also noted in the same region. Pulses are palpable. Pain is present during both passive abduction and adduction of the fingers. Compartment pressure, measured using a Stryker needle, is found to be 42 mmHg.

**Table 2:** Methodology overview for evaluating language models.

Step	Details
1. Vignette Creation	Created 30 patient scenarios based on common plastic surgery emergencies (e.g., hand surgery, burns, facial fractures). Initially excluded physical exam details, then enhanced with physical exam information.
2. Design Prompts	Formulated separate prompts for diagnosis and management tasks.
3. Initial Prompt	Presented vignettes without physical exam details to each LLM (ChatGPT-4 and Gemini) for diagnostic interpretation in separate sessions.
4. Second Prompt	Reintroduced vignettes with physical exam details to each LLM, seeking management advice in separate sessions.
5. Record Responses	Recorded all LLM responses. Responses scored on a three-point Likert scale by three independent medical professionals against standards from ‘*Plastic Surgery Emergencies: Principles and Techniques, Second Edition’*.
6. Distribution Analysis	Categorized the frequency of LLM responses based on their Likert scores (1, 2, 3) for both diagnosis and management tasks (with/without physical exam data).
7. Statistical Analysis	Calculated performance metrics (median, mode) for each category (diagnosis and management, with/without physical exam). Employed the Wilcoxon rank-sum test to assess differences in model performance, with significance set at *p* < 0.05.

**Table 3:** Descriptive statistics of the large language models’ performance in delivering diagnosis and management.

LLMs	Category	Score 1 (W/O PE)	Score 1 (W/PE)	Score 2 (W/O PE)	Score 2 (W/PE)	Score 3 (W/O PE)	Score 3 (W/PE)	Median	Mode
ChatGPT-4	Diagnosis	3 (10%)	0 (0%)	3 (10%)	3 (10%)	24 (80%)	27 (90%)	W/PE = 3.0 W/O PE = 3.0	W/PE = 3 W/O PE = 3
	Management	1 (3.33%)	0 (0%)	22 (73.33%)	22 (73.33%)	7 (23.33%)	8 (26.67%)	W/PE = 2.0 W/O PE = 2.0	W/PE = 2 W/O PE = 2
Gemini	Diagnosis	8 (26.67%)	4 (13.33%)	6 (20.00%)	6 (20.00%)	16 (53.33%)	20 (66.67%)	W/PE = 3.0 W/O PE = 3.0	W/PE = 3 W/O PE = 3
	Management	5 (16.67%)	7 (23.33%)	22 (73.33%)	19 (63.33%)	3 (10.00%)	4 (13.33%)	W/PE = 2.0 W/O PE = 2.0	W/PE = 2 W/O PE = 2

LLMs (large language models); W/PE (with a physical exam); W/O PE (without a physical exam).

**Table 4:** Analysis of the large language models’ performance across different types of vignettes.

Comparison	Wilcoxon W	*p*-Value	Accuracy % (W/O PE)	Accuracy % (W/PE)
ChatGPT-4 Diagnosis W/O vs. W/PE	400.5	0.24	90	100
ChatGPT-4 Management W/O vs. W/PE	424	0.62	96.67	100
Gemini Diagnosis W/O vs. W/PE	378	0.23	73.33	86.67
Gemini Management W/O vs. W/PE	464.5	0.8	83.33	76.67

W/PE (with a physical exam); W/O PE (without a physical exam).

**Table 5:** Comparative analysis of the large language models’ performance.

Comparison	Wilcoxon W	*p*-Value
ChatGPT-4 vs. Gemini Diagnosis W/O PE	573	0.030
ChatGPT-4 vs. Gemini Diagnosis W/PE	561	0.023
ChatGPT-4 vs. Gemini Management W/O PE	554	0.048
ChatGPT-4 vs. Gemini Management W/PE	587	0.014

W/PE (with a physical exam); W/O PE (without a physical exam).

## Data Availability

The original contributions presented in the study are included in the article; further inquiries can be directed to the corresponding author.

## References

[1] Singh H., Meyer A.N.D., Thomas E.J. (2014). The frequency of diagnostic errors in outpatient care: Estimations from three large observational studies involving US adult populations. BMJ Qual. Saf..

[2] Gunderson C.G., Bilan V.P., Holleck J.L., Nickerson P., Cherry B.M., Chui P., Bastian L.A., Grimshaw A.A., Rodwin B.A. (2020). Prevalence of harmful diagnostic errors in hospitalised adults: A systematic review and meta-analysis. BMJ Qual. Saf..

[3] Newman-Toker D.E., Wang Z., Zhu Y., Nassery N., Tehrani A.S.S., Schaffer A.C., Yu-Moe C.W., Clemens G.D., Fanai M., Siegal D. (2021). Rate of diagnostic errors and serious misdiagnosis-related harms for major vascular events, infections, and cancers: Toward a national incidence estimate using the “Big Three”. Diagnosis.

[4] Newman-Toker D.E., Schaffer A.C., Yu-Moe C.W., Nassery N., Tehrani A.S.S., Clemens G.D., Wang Z., Zhu Y., Fanai M., Siegal D. (2019). Serious misdiagnosis-related harms in malpractice claims: The “Big Three”—Vascular events, infections, and cancers. Diagnosis.

[5] McDuff D., Schaekermann M., Tu T., Palepu A., Wang A., Garrison J., Singhal K., Sharma Y., Azizi S., Kulkarni K. (2023). Towards accurate differential diagnosis with large language models. arXiv.

[6] Shimkhada R., Solon O., Tamondong-Lachica D., Peabody J.W. (2016). Misdiagnosis of obstetrical cases and the clinical and cost consequences to patients: A cross-sectional study of urban providers in the Philippines. Glob. Health Action.

[7] Graber M.L., Wachter R.M., Cassel C.K. (2012). Bringing diagnosis into the quality and safety equations. JAMA.

[8] Choi I., Lee S.-M., Flynn L., Kim C.-M., Lee S., Kim N.-K., Suh D.-C. (2016). Incidence and treatment costs attributable to medication errors in hospitalized patients. Res. Social. Adm. Pharm..

[9] Gandhi T.K., Kachalia A., Thomas E.J., Puopolo A.L., Yoon C., Brennan T.A., Studdert D.M. (2006). Missed and delayed diagnoses in the ambulatory setting: A study of closed malpractice claims. Ann. Intern. Med..

[10] Schiff G.D., Hasan O., Kim S., Abrams R., Cosby K., Lambert B.L., Elstein A.S., Hasler S., Kabongo M.L., Krosnjar N. (2009). Diagnostic error in medicine: Analysis of 583 physician-reported errors. Arch. Intern. Med..

[11] Car L.T., Papachristou N., Bull A., Majeed A., Gallagher J., El-Khatib M., Aylin P., Rudan I., Atun R., Car J. (2016). Clinician-identified problems and solutions for delayed diagnosis in primary care: A PRIORITIZE study. BMC Fam. Pract..

[12] Wang F., Luo W. (2005). Assessing spatial and nonspatial factors for healthcare access: Towards an integrated approach to defining health professional shortage areas. Health Place.

[13] Hub R.H.I. (2024). Healthcare Access in Rural Communities. https://www.ruralhealthinfo.org/topics/healthcare-access.

[14] Hasan M., Fukuda A., Maruf R.I., Yokota F., Ahmed A. Errors in remote healthcare system: Where, how and by whom?. Proceedings of the TENCON 2017—2017 IEEE Region 10 Conference.

[15] Association A.H. (2024). How AI Is Improving Diagnostics, Decision-Making and Care. https://www.aha.org/aha-center-health-innovation-market-scan/2023-05-09-how-ai-improving-diagnostics-decision-making-and-care.

[16] Gomez-Cabello C.A., Borna S., Pressman S., Haider S.A., Haider C.R., Forte A.J. (2024). Artificial-Intelligence-based clinical decision support systems in primary care: A scoping review of current clinical implementations. Eur. J. Investig. Health Psychol. Educ..

[17] Secinaro S., Calandra D., Secinaro A., Muthurangu V., Biancone P. (2021). The role of artificial intelligence in healthcare: A structured literature review. BMC Med. Inform. Decis. Mak..

[18] Bajwa J., Munir U., Nori A., Williams B. (2021). Artificial intelligence in healthcare: Transforming the practice of medicine. Future Healthc. J..

[19] Kitsios F., Kamariotou M., Syngelakis A.I., Talias M.A. (2023). Recent advances of artificial intelligence in healthcare: A systematic literature review. Appl. Sci..

[20] Gholipour M., Khajouei R., Amiri P., Gohari S.H., Ahmadian L. (2023). Extracting cancer concepts from clinical notes using natural language processing: A systematic review. BMC Bioinform..

[21] OpenAI (2024). ChatGPT-4 [Large Language Model]. https://chat.openai.com.

[22] AI G. (2024). Gemini [Large Language Model]. https://gemini.google.com/u/0/app.

[23] Wang S., Zhao Z., Ouyang X., Wang Q., Shen D. (2023). Chatcad: Interactive computer-aided diagnosis on medical image using large language models. arXiv.

[24] Reese J.T., Danis D., Caufield J.H., Groza T., Casiraghi E., Valentini G., Mungall C.J., Robinson P.N. (2023). On the limitations of large language models in clinical diagnosis. medRxiv.

[25] Park Y.-J., Pillai A., Deng J., Guo E., Gupta M., Paget M., Naugler C. (2024). Assessing the research landscape and clinical utility of large language models: A scoping review. BMC Med. Inform. Decis. Mak..

[26] Sallam M. (2023). The utility of ChatGPT as an example of large language models in healthcare education, research and practice: Systematic review on the future perspectives and potential limitations. medRxiv.

[27] Kwon T., Ong K.T.-I., Kang D., Moon S., Lee J.R., Hwang D., Sim Y., Sohn B., Lee D., Yeo J. (2024). Large language models are clinical reasoners: Reasoning-aware diagnosis framework with prompt-generated rationales. Proc. AAAI Conf. Artif. Intell..

[28] Iannantuono G.M., Bracken-Clarke D., Floudas C.S., Roselli M., Gulley J.L., Karzai F. (2023). Applications of large language models in cancer care: Current evidence and future perspectives. Front. Oncol..

[29] Warrier A., Singh R., Haleem A., Zaki H., Eloy J.A. (2024). The comparative diagnostic capability of large language models in otolaryngology. Laryngoscope.

[30] Mehnen L., Mehnen L., Gruarin S., Vasileva M., Knapp B. (2023). ChatGPT as a medical doctor? A diagnostic accuracy study on common and rare diseases. medRxiv.

[31] Krusche M., Callhoff J., Knitza J., Ruffer N. (2024). Diagnostic accuracy of a large language model in rheumatology: Comparison of physician and ChatGPT-4. Rheumatol. Int..

[32] Bullocks J.M., Bullocks J.M., Hsu P.W., Izaddoost S.A., Hollier L. (2017). Plastic Surgery Emergencies: Principles and Techniques.

[33] Barnard F., Van Sittert M., Rambhatla S. (2023). Self-diagnosis and large language models: A new front for medical misinformation. arXiv.

[34] Isabel (2024). Isabel pro Differential Diagnosis Generator. https://www.isabelhealthcare.com.

[35] Balas M., Ing E.B. (2023). Conversational AI models for ophthalmic diagnosis: Comparison of chatgpt and the isabel pro differential diagnosis generator. JFO Open Ophthalmol..

[36] Mello M.M., Guha N. (2023). ChatGPT and physicians’ malpractice risk. JAMA Health Forum.

[37] Garg R.K., Urs V.L., Agarwal A.A., Chaudhary S.K., Paliwal V., Kar S.K. (2023). Exploring the role of ChatGPT in patient care (diagnosis and treatment) and medical research: A systematic review. Health Promot. Perspect..

